# LONELINESS AND ASSOCIATED OUTCOMES IN A LARGE SURVEY OF OLDER ADULTS

**DOI:** 10.1093/geroni/igz038.659

**Published:** 2019-11-08

**Authors:** Timothy Barnes, Gandhi Bhattarai, Sandra Kraemer, Shirley Musich, James Schaeffer, Charlotte S Yeh

**Affiliations:** 1 Optum, Ann Arbor, Michigan, United States; 2 UnitedHealthcare, Minneapolis, Minnesota, United States; 3 AARP Services, Inc., Washington, District of Columbia, United States

## Abstract

Loneliness and social isolation are major risk factors for poor physical and mental health among older adults. Studies demonstrate that loneliness is associated with depression, impaired cognitive performance, increased chronic disease, and mortality. However, the impact on other psychosocial constructs and healthcare outcomes remain understudied. The purpose of this study was to estimate the prevalence of loneliness and examine associations with socio-demographic, medical, and psychosocial characteristics in a large national survey of older adults (N=4,525). Overall, 43% of participants reported either moderate or severe loneliness. Older age, female gender, income, depression, hearing difficulty, and poorer health were all associated with loneliness. Purpose of life, resilience, optimism, and a diverse social network were associated with low loneliness. On average, lonely participants had a higher rate of emergency department visits, inpatient admissions, and medical costs. Based on findings, interventions should aim to alleviate loneliness among older adults.

